# Liquid chromatography-mass spectrometry approach for characterizing sucrose isomers in complex mono-floral honey

**DOI:** 10.1007/s00216-025-05988-9

**Published:** 2025-07-09

**Authors:** Enoch Amoah, Santosh Raman Acharya, Ayesha Seth, Abraham K. Badu-Tawiah

**Affiliations:** Department of Chemistry and Biochemistry, The Ohio State University, 100 W. 18th Avenue, Columbus, OH 43210 USA

**Keywords:** Mono-floral honey, Sucrose isomers, Microdroplet chemistry, Analytical methods, Liquid chromatography-mass spectrometry

## Abstract

Sucrose, which forms < 2% of the chemical content in honey samples, is known to have five structural isomers each with its own medicinal benefits. Unfortunately, studies characterizing the specific sucrose isomer(s) present in honey samples are limited. Herein, we introduce a contained electrospray ionization (cESI) method that can be coupled between liquid chromatography (LC) and tandem mass spectrometry (MS/MS). This LC-cESI-MS/MS platform leverages chloride adduction to enable sensitive differentiation and characterization of disaccharide isomers in complex honey samples. By integrating retention time and collision-induced dissociation (CID) MS/MS data, we achieved orthogonal analysis of six sucrose isomers. The MS/MS on the chloride adducts showed distinct fragment ions for each isomer. Additional optimization afforded nanomolar (nM) detection limits for all disaccharides analyzed via chloride adduction in negative-ion mode, a feature that showed superior sensitivity compared with conventional sodium adduction methods typically achieved in positive-ion mode. We identified four sucrose isomers (turanose, palatinose, maltulose, and trehalulose) in three mono-floral honey samples, of which turanose was the most abundant isomer. Sucrose itself could not be confirmed in any of the honey samples tested and leucrose was confirmed to be absent. Although the specific amounts of these isomers were not determined, principal component analysis showed that the abundances of the four identified structural isomers significantly differed in the three mono-floral honey samples. The current study forms the first report suggesting turanose to be the main sucrose isomer in the tested mono-floral honey. Such identification was made possible because of our ability to independently optimize LC and cESI spray solvents, and to enable online microdroplet chemistry via chloride adduction, which allowed the conventional CID-MS/MS to yield highly informative fragmentation.

**Graphical Abstract:**

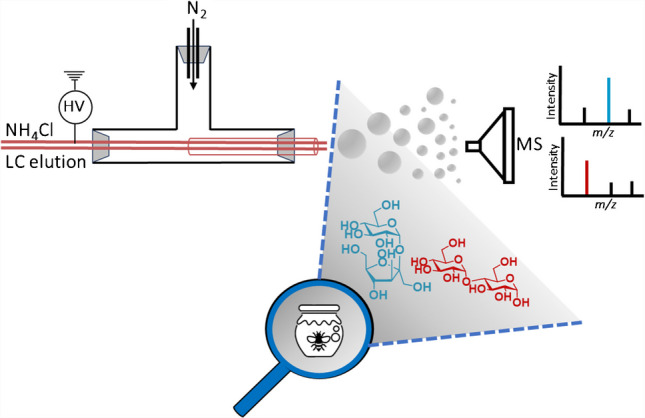

**Supplementary Information:**

The online version contains supplementary material available at 10.1007/s00216-025-05988-9.

## Introduction

**Fig. 1:**
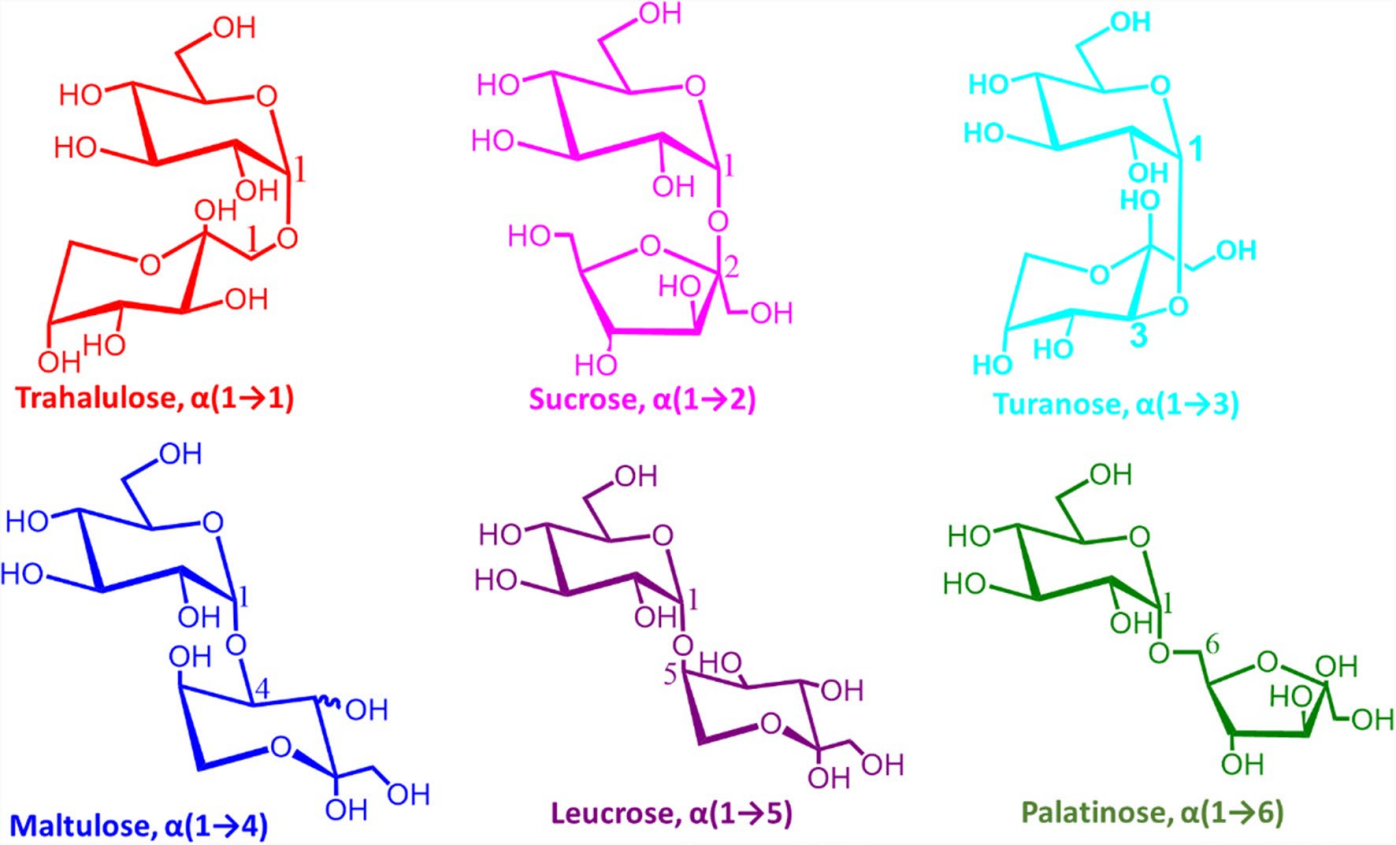
Structures of positional isomers of sucrose

Honey presents a rich source of natural macro- and micro-nutrients, of which fructose and glucose form the major components (70%). A wide range of minor constituents in honey includes maltose, sucrose, higher-order carbohydrates, polyphenols, vitamins, and minerals [1]. Interestingly, the actual composition of honey is dependent on several factors, including the floral source used, seasonal, and environmental [2]. These factors have led to more than 300 varieties of honey worldwide, each with its unique color, flavor, and biological effects. For example, one of the key distinctions is whether the honey is mono- or poly-floral. Mono-floral honey is the type of honey that is predominantly prepared from the nectar of a single plant species, of which some 40 different types are known [3]. While various analytical methods have been developed to analyze the main sugar contents in honey (as well as to detect specific organic compounds) [4–7], the analysis of isomeric sugars directly from honey is limited. It is possible to isolate nucleic acids from plant pollen in honey to obtain botanical source/information [8], but the ability to detect the enrichment of specific sugar isomers in specific honey samples can be extremely useful in a variety of ways, including the potential isolation of rare sugars [9, 10]. For this reason, we sought to study the composition of sucrose isomers in three mono-floral honey samples (i.e., Rewarewa, Manuka, and Tawari), all produced in New Zealand. We are interested in the five structural isomers of sucrose, which are made up of the same monosaccharide units (fructose and glucose) but possess only 50% of the sweetness of sucrose. The distinct health benefits of these sucrose isomers are well-known, yet they differ only in the position of the glycosidic bond (Fig. 1) [11–15]. This slight difference at the glycosidic bond offers many different bioactivities, including turanose α(1 → 3), which is capable of impeding lipid accumulation [16]. Palatinose α(1 → 6) is reported to reduce insulinemic response [13, 17]. Therefore, orthogonal analytical methods sensitive to small changes in chemical structure of saccharides and capable of handling smaller sample volumes while also offering quantitative capabilities in a short amount of time are highly desirable.

For years, advancements in the analysis of saccharides lagged that for other biomolecules such as proteins and nucleic acids. Nuclear magnetic resonance (NMR) is among the few techniques that have been efficient at saccharide isomer differentiation. However, NMR requires pure and milligram quantities of samples [6, 18, 19]. Separation techniques such as gas chromatography (GC) provide adequate means of differentiating saccharide isomers, but due to low volatility and hydrophilicity, conversion of the sugars into stable derivatives is required to achieve satisfactory results [6, 20, 21]. This requirement makes saccharide analysis tedious and time-consuming using GC. Ion mobility spectrometry (IMS) is a gas-phase separation technique that offers seamless combination with MS. IMS can separate saccharide ions by shape, size, and charge, and allows isomer resolution based on collision cross-section prior to MS analysis. However, IMS is not available on ordinary mass spectrometers, limiting their widespread utility [22]. In this regard, liquid chromatography (LC) can provide a front-end separation method for ordinary mass spectrometers. When coupled with mass spectrometry (MS), [23] LC–MS serves as a powerful tool for obtaining the necessary orthogonal information on retention time, governed by preferential interactions with specific stationary phases and molecular weight for elemental composition (i.e., molecular formula). For complex mixtures where co-elution is highly possible, a third analytical information in the form of tandem MS (MS/MS) analysis is necessary to gain insight into the connectivity of atoms identified from the molecular formula to aid in complete characterization and differentiation of saccharide isomers. Energy-resolved (ER) MS has also proven useful in isomer differentiation when collision-induced dissociation (CID)-MS/MS is performed as a function of collision energy [24]. However, performing ER-MS on chromatographically eluting species might be challenging because of the multiple collision energies required for a specific species.

Without the front-end separation, MS has shown high sensitivity and specificity for detection and structural characterization of saccharides [25–37]. This was made possible through the development of ion activation techniques that allowed a more efficient fragmentation of saccharides through cross-ring and glycosidic bond cleavages, including electron capture dissociation, infrared dissociation as well as ultraviolet dissociation [38–46]. CID is the most common ion activation method for MS/MS available on almost all mass spectrometers for structural characterization. Nonetheless, challenges persist for CID-MS/MS in terms of its capacity to differentiate closely related saccharide isomers, like stereoisomers. In such instances, a front-end separation step can provide complementary information to enable complete identification of isomers via CID fragmentation. Even more interesting would be an approach that can empower the conventional CID ion activation method to facilitate deep fragmentation involving both glycosidic and cross-ring cleavages. Such method will enable widespread use of ordinary mass spectrometers offering obvious advantages over NMR, including rapid analysis, sensitivity, and the use of unpurified samples.

Recently, our research group employed a direct infusion mass spectrometry approach to characterize and differentiate isomers of sucrose using halide adduction in complex mixtures, in which we identified four of the structural isomers of sucrose in four different brands of honey samples [31]. In a related study, we used microdroplet-based phenylboronic acid chemistry to enable ultra-sensitive detection of saccharides in electrospray ionization (ESI)–MS [47]. While the phenylboronic acid chemistry was subsequently employed in LC-CID-MS/MS via online derivatization of sugars to successfully differentiate isobaric disaccharide isomers in complex mixtures [48], it was nontrivial to achieve both sensitive detection and isomer differentiation in the same experiment. This was due to multiple products (e.g., mono- and di-substituted phenylboronate esters) derived from the microdroplet-based derivatization process, which reduced sensitivity.

Therefore, the main objective of the present study is to couple LC to CID-MS/MS analysis via online halide adduction to enable highly sensitive detection of disaccharides while also enabling the differentiation of positional isomers in complex mixtures. The attachment of anion such as halides is a well-known mechanism for negative ions formation as demonstrated earlier by Cole and co-workers [49, 50]. Exposure of polar functional groups (in this case OH in sugars) to halides makes adduct formation highly feasible. Chloride anions were chosen for this study because it can easily form stable adduct and provide abundant diagnostic fragment ions when the adduct is subjected to CID activation [31]. Adduct formation was facilitated online in the microdroplet environment using contained electrospray ionization (cESI) source [51–53], which uses co-axial spray to achieve instantaneous derivatization of analytes eluting from the LC column. The novelty of our LC-cESI-MS/MS method is in our ability to independently optimize the LC and the electrospray conditions and thus providing a true orthogonal experimental design to resolve closely related isomers. Specifically, without addition ammonium chloride reagent to the mobile phase, we are able to use high concentrations (5 mM) to instantaneously modify the LC eluent during the stages of microdroplet formation in cESI, which allowed high ion yields. This approach enabled us to automate microdroplet chemistry with ease and to do so in a high throughput fashion with high sensitivity. While few studies have employed halide adduction in saccharide analysis [49, 54–56], its application to acquire true orthogonal results for differentiation and characterization of isomers with higher sensitivity on an LC–MS/MS format has not been demonstrated.

## Experimental section

### Contained electrospray ionization platform

**Fig. 2:**
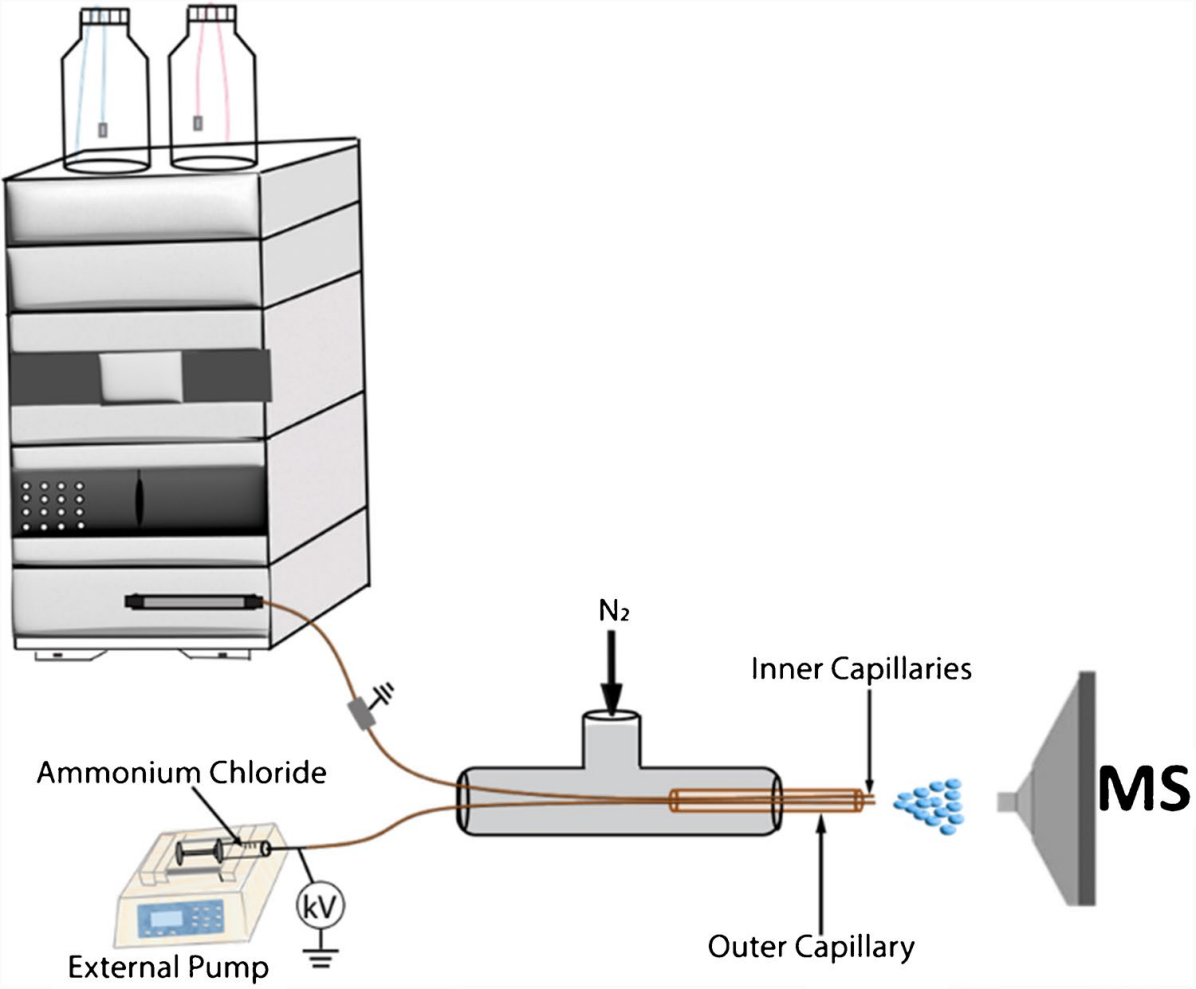
Experimental setup for LC-cESI-MS/MS, utilizing a co-axial contained electrospray source for online modification of sugars eluting from LC for instantaneous MS/MS analysis

Figure 2 shows a schematics of the co-axial cESI platform coupled with high performance liquid chromatography (HPLC). Eluent from the HPLC is instantaneously modified by the cESI source under the microdroplet environment and transferred to the proximal mass spectrometer as shown in Fig. 2. As described in other publications from our group [47, 48, 51–53, 57], the cESI platform is created from a stainless-steel Tee with compression fittings. The reagent solution and the eluent from the HPLC are delivered through two separate inner silica capillaries of internal diameter (ID)100 µm extending from a larger fused silica outer capillary of 450 µm ID. A direct current (DC) high voltage (3.0 kV optimized) is applied to the inner capillary from which the modifying reagent is delivered. Here, our reagent is ammonium chloride serving as the source of chloride ions. As the analytes elute from the column, they pass through a PEEK tubing of ID 0.004 inches and are exposed to the chloride ions where attack of the hydroxyl group of the sugars is initiated by the Cl^–^ to form adducts in the electrosprayed microdroplets. The HPLC electronics are shielded from the applied DC high voltage by installing a grounded stainless-steel coupler between the cESI source and the HPLC. Nitrogen sheath gas (70 PSI) is conveyed to the ion source to aid microdroplet formation, mixing, and desolvation.

### Liquid chromatography-tandem mass spectrometry

An Agilent 1100 HPLC system (Agilent Technologies, Santa Clara, CA) coupled to Thermo Fisher Scientific Velos Pro ion trap mass spectrometer (San Jose, CA, USA) was employed for the separation and detection of saccharide adducts. Waters Acquity BEH Amide column, 100 mm × 2.1 mm, 1.7 µm (Waters Corp., Milford, MA) was used for the chromatographic separation of the sugars in an isocratic condition of 84:16 (v/v) acetonitrile/water with 0.1% (v/v) ammonium hydroxide mobile phase at a flow rate of 130 µL/min. The addition of ammonium hydroxide as a modifier was necessary to mitigate peak splitting/broadening that can be encountered with reducing sugars due to anomeric mutarotation [58]. In addition, ammonium hydroxide as a modifier serves to improve retention [47]. All analyses were performed using 1 µL sample injection volume. Data acquisition and processing were done using Thermo Fisher Scientific Xcalibur 2.2 SP1 software at the following MS parameters: 3 microscans, 100 ms ion injection time, and 350 °C inlet capillary temperature. A constant distance of 5 mm was maintained between the cESI platform and the mass spectrometer inlet. Identification and characterization of sugar isomers were achieved using retention time and tandem MS with CID at normalized collision energy of 30% (manufacturer’s unit) with 1.5 Th (mass/charge units) isolation window. The entire analysis time was 35 min.

### Chemicals and reagents

Sucrose (≥ 99.5%), ^13^C_12_-Sucrose (≥ 99%), turanose (≥ 98%), maltulose (≥ 99.3%), leucrose (≥ 98%), and palatinose (≥ 98%) were purchased from Sigma-Aldrich (St. Louis, MO). Trehalulose (≥ 98%) was ordered from Biosynth International, Inc (San Diego CA). LCMS-grade acetonitrile and ammonium chloride (NH_4_Cl)/hydroxide (NH_4_OH) were purchased from Acros Organics (NJ, USA) and Sigma-Aldrich (St. Louis, MO), respectively.

### Preparation of standard sugar solutions

Individual sugar standard stock solutions (~ mM) were prepared in MilliQ water. An aliquot of each standard solution was dissolved in acetonitrile/water (75:25, v/v) to form a standard solution of 2 µM for analyses. For analysis of the mixture, an aliquot of each individual standard stock solution was diluted in acetonitrile/water (75:25, v/v) to a final concentration of 2 µM for eachsugar.

### Preparation of honey samples

Three different high-grade mono-floral honey samples, namely Manuka, Rewarewa, and Tawari, were obtained from Manawa honey, New Zealand. The honey samples (50 mg/mL) were prepared by diluting 500 mg of each honey sample in 10 mL of MilliQ water. Then, 5 mL of diluted samples were centrifuged using a Microsep™ Advance 3 k MW cutoff Centrifugal Filter (Pall Corp., Ann Arbor, MI) at ~ 3000 rpm for 1 h. Finally, 250 µg/mL of each of the filtered honey samples in acetonitrile/water (75:25, v/v) was analyzed.

### Preparation of calibration samples

A series of the standard disaccharide isomer solutions (0.1, 0.2, 0.3,0.5, 0.75, and 1.0 μM) were prepared in acetonitrile/water (75:25, v/v). ^13^C_12_-Sucrose (0.5 μM) was used as an internal standard, which was added to each standard solution. Analyses of the final solutions were performed in the negative-ion mode using the LC-cESI-MS/MS, except for the comparative study of the isomers in positive-ion mode, where the commercial HESI source was employed. The analysis of each calibration solution was performed in six replicates.

## Results and discussion

### Process optimization

To obtain efficient sugar-chloride adduct formation, it was necessary to optimize the flow rate and concentration of the NH_4_Cl reagent, as well as the spray voltage. The optimization was performed using the cESI platform in direct infusion MS format. In this case, sucrose (100 µM in Acetonitrile:Water, 1:1, v/v) was used as a representative sugar, which was introduced via one of the inner capillaries of the cESI platform while the NH_4_Cl reagent (1 mM) was introduced via a second capillary from an external pump (at 20 µL/min). We first optimized the voltage to achieve optimum Cl^–^ ion generation in microdroplets. As shown in Fig. S1, we found that the formation of the adduct at *m/z* 377 increased as the voltage was increased from 1.5 kV to an optimum voltage of 3.0 kV, where the ion intensity began to decrease accordingly upon further increase in voltage. As a result, a voltage of 3.0 kV was chosen for final analysis. In the optimization of reagent flow rate, our goal was to increase the formation of chloride-sugar adduct (*m/z* 377) while minimizing the formation of the deprotonated sugar (*m/z* 341) to achieve higher sensitivity. The NH_4_Cl reagent flow rate was varied from 5 µL/min to 60 µL/min with varying voltage, as shown in Fig. S2. We varied both the flow rate and the applied voltage to validate the observations made during the voltage optimization phase and to assess whether flow rate influenced the system’s response under varying voltage conditions. At lower flow rates of 5 and 10 µL/min, we found that the deprotonated sugar dominated the formation of chloride–sugar adducts. Beyond a flow rate of 10 µL/min, we observed that the formation of chloride–sugar adducts increased with increasing flow rate to an optimum flow rate of 40 µL/min. It can also be observed that the ion intensity decreases with increasing voltage at a flow rate above 10 µL/min. Hence, an optimum flow rate of 40 µL/min was chosen for further experiments. In this later experiment, we subsequently optimized the NH_4_Cl reagent concentration at a flow rate of 40 µL/min and a voltage of 3.0 kV. We found that the chloride–sugar adduct ion intensity increased with increasing NH_4_Cl concentration, until a maximum of 5 mM before it began to decrease (Fig. S3). Additionally, we found that nitrogen sheath gas pressure of 70 PSI provided optimal conditions for adduct formation (data not shown). Therefore, all subsequent experiments were conducted at the following optimized conditions: voltage at 3.0 kV, reagent flow rate at 40 µL/min, NH_4_Cl reagent concentration of 5 mM, nitrogen sheath gas flow at 70 PSI, and capillary temperature at 350 °C. In all cases, the sucrose analyte was introduced from the main capillary at a flow rate of 130 µL/min in these direct infusion experiments to mimic the flow rate from the HPLC.

### LC-cESI-MS/MS analysis of sugars

Although research from our group and others has reported interesting results in carbohydrate chemistry, the analysis of isomeric sugars with MS alone remains challenging, especially in a complex mixture. Likewise, the separation of isomeric sugars in a complex mixture using LC alone is nontrivial due to co-elution in some instances, leading to insufficient resolution. We believe that there is an opportunity to couple LC to MS/MS via the conventional CID technique, which will enable ordinary mass spectrometers to provide useful and reliable fragmentation information that can facilitate the differentiation of isomeric sugars in complex mixtures. In this study, we separated the isomers using hydrophilic interaction liquid chromatography (HILIC) technique known for its ability to resolve highly polar compounds like sugars [58–61]. In a previous LC–MS method designed to improve the sensitivity gain of sugars using phenyl boronic acid reaction, we found that isocratic elution using acetonitrile/water provided the best separation of isobaric disaccharides [48]. However, unlike the phenylboronic acid reagent, we expected the online chloride adduction to offer high sensitivity due to the formation of a single predominant ion, as well as to provide diagnostic fragment ions in CID MS/MS.

In a typical experiment, we exposed the disaccharide isomers eluting from the chromatographic column to a Cl^–^ source in real time using the cESI platform to enable efficient sugar ionization at low concentrations. The cESI platform is advantageous in that it enables us to independently optimize the LC and ion source conditions separately to achieve highly sensitive analysis. Here, we combine retention time and CID MS/MS data to achieve true orthogonal results that enable efficient characterization of sucrose isomers in complex mixtures. Since the disaccharides have a molecular weight of 342 Da and the mass of chloride anion is 35 Da, we expected to observe successful adduction at *m/z* 377 representing chloride-sugar adduct [M + Cl]^–^. As a result, the experiment was performed in negative-ion mode at *m/z* 377 product ion scan. The distinctive chloride isotopes ^35^Cl:^37^Cl with an abundance of 3:1 were also useful in the full mass in determining important peaks.

Following the successful optimization of the various experimental conditions, the method was applied to the analysis of the mixture of standard as well as individual disaccharide isomers differing only in their glycosidic bonds, namely trehalulose α(1 → 1), sucrose α(1 → 2), turanose α(1 → 3), maltulose α(1 → 4), leucrose α(1 → 5), and palatinose α(1 → 6). To achieve efficient separation in a reasonable time, we first studied the effect of LC flow rate and column temperature on retention time. Figure S4 shows the chromatograms at various LC flow rates of 130, 140, and 150 μL/min. We found that the flow rate at 150 μL/min provided the best results among the three with respect to effects on overall analysis time. We further studied the effect of column temperature on retention time and hence overall analysis time. Figure S5 represents the chromatograms for three different column temperatures at 38, 45, and 55 °C performed at the optimized flow rate of 150 μL/min. In this instance, we observed that increasing temperature resulted in a significant decrease in retention time and hence improvement in overall analysis time. Hence, all other analyses were performed at an LC flow rate of 150 μL/min and column temperature of 55 °C.

**Fig. 3:**
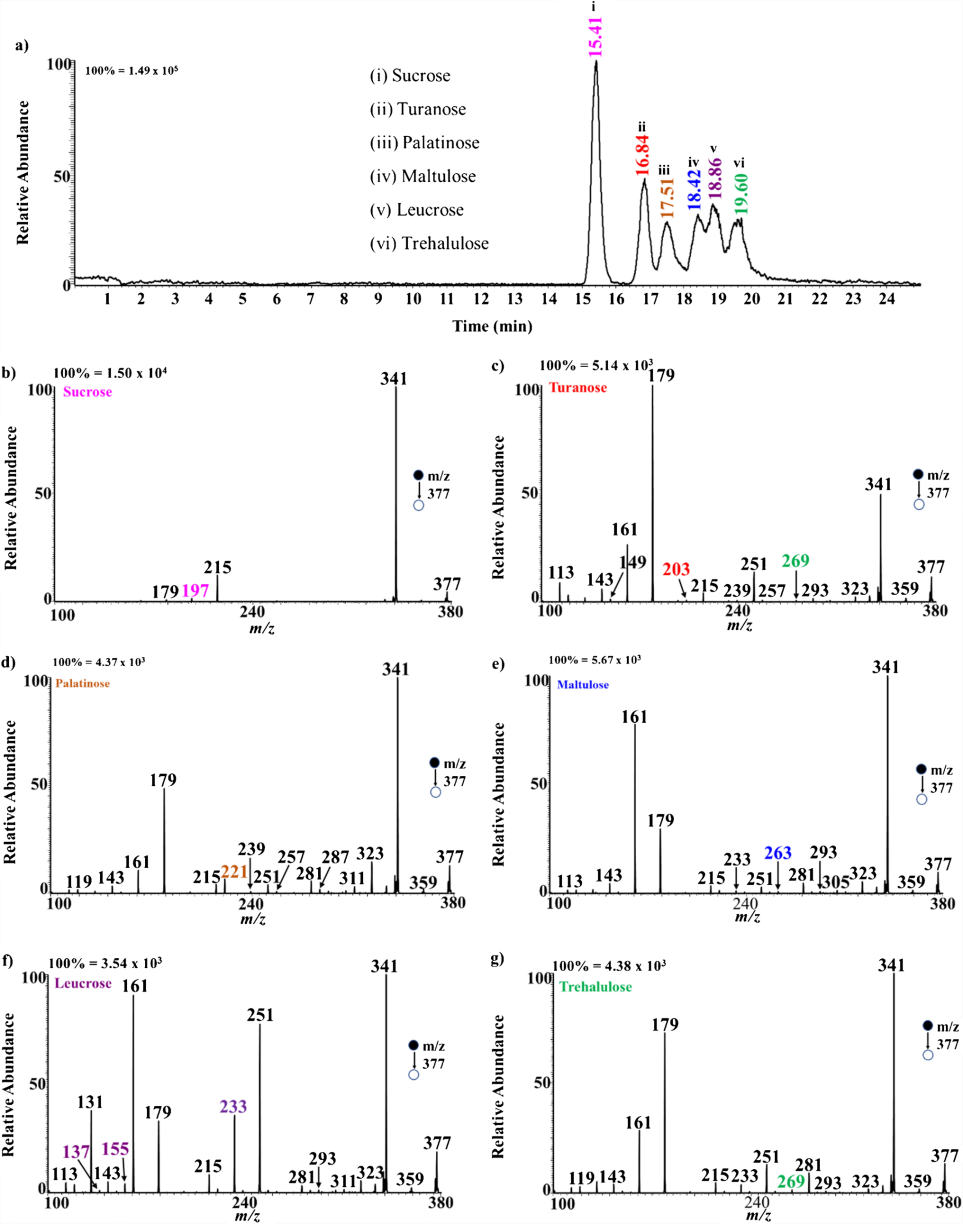
**a** Chromatogram generated using LC–MS/MS product ion scanning with chloride adduction. **b**–**g** Negative-ion mode product ion spectra for the isomers of sucrose (2 µM) in the standard chromatogram, all at *m/z* 377. When prepared in a mixture, the isomers were consistently observed to elute earlier than when the isomers were injected as individual standards

Figure 3a shows the chromatogram generated for a mixture of sucrose and its five isomers using LC–MS/MS. Though peak broadening is still observed in the chromatogram, satisfactory separation (peak-to-peak) is achieved for the six positional isomers. The chloride adduction of the disaccharides provides sensitive detection at a relatively lower sample concentration of 2 µM. To obtain a two-dimensional data based on retention time and diagnostic fragment ions to enhance confidence in the results, CID fragmentation was performed on *m/z* 377 at each retention time. Figure 3b–g show the tandem MS spectra for each chromatographic peak from which diagnostic ions were obtained for each sugar, with diagnostic ions highlighted in different colors. For example, tandem MS analysis of the peak at retention time 15.41 min for sucrose shows that the disaccharide loses HCl from *m/z* 377 (chloride-sugar adduct) to form a highly intense peak at *m/z* 341 (Fig. 3b), representing the deprotonated sucrose, [M-H]^–^. Sucrose was also observed to undergo a neutral loss of C_6_H_10_O_5_ (MW 162 Da) via glycosidic bond breakage to form *m/z* 215, which subsequently loses H_2_O to form a diagnostic ion at *m/z* 197. This is very consistent with previous studies conducted using nano-electrospray ionization via direct infusion MS [31]. Tandem MS analyses of all other peaks were observed to fragment in similar pattern as sucrose but with distinct diagnostic fragment ions. The MS/MS of turanose α (1 → 3 linkage) shows a neutral loss of HCl from the adduct to form *m/z* 341 (Fig. 3c). It also shows other major peaks at *m/z* 251 due to the loss of C_3_H_6_O_3_ (MW 90 Da) from *m/z* 341 via glycosidic bond cleavage. The peak at *m/z* 203 is observed as a result of the loss of CH_2_O (MW 30 Da) and H_2_O (MW 18 Da) from the ion at *m/z* 251. Another peak at *m/z* 179 is due to the neutral loss of C_6_H_10_O_5_ via glycosidic bond cleavage from the deprotonated sugar. This can undergo a subsequent loss of H_2_O to form the ion at *m/z* 161, which further loses H_2_O to form *m/z* 143. Loss of CH_2_O from *m/z* 161 forms the ion at *m/z* 131. All other isomers, namely maltulose α (1 → 4 linkage), leucrose α(1 → 5 linkage), and palatinose α(1 → 6 linkage), were found to fragment in a similar manner as turanose but at a relatively different intensities. Comparing the entire MS profile of the isomers, we obtained diagnostic ions for each of the other isomers, as summarized in Table 1. For example, leucrose produced three unique diagnostic ions at *m/z* 233, 155, and 137. On the other hand, turanose fragmented to provide one unique diagnostic ion at *m/z* 203, which is absent in the other isomers. Likewise, palatinose, maltulose, and trehalulose produced diagnostic ions at *m/z* 221, 263, and 269, respectively. The observed differences in tandem MS spectra are primarily attributed to variations in cross-ring and glycosidic bond breakages during CID. The main difference between the isomers analyzed is in their glycosidic linkage positions (e.g., sucrose α(1 → 2) vs. palatinose α(1 → 6)) and yet distinct fragment ions are observed when the chloride adducts are activated via collisions. In all cases, HCl is lost first, followed by unique cross-ring and glycosidic bond cleavages. The cascade of dissociations leads to different diagnostic ions for each isomer as described in Table 1. The use of chloride adducts in negative-ion mode is advantageous over the conventional Na^+^ adducts in positive-ion mode because Na^+^ adducts fragment only through glycosidic bond cleavages, which limits their ability to differentiate linkage isomers [62, 63]. We propose that this limited fragmentation is due to the high binding capacity of Na^+^ toward saccharides, which leads to a less mobile Na^+^ ion with fewer charged centers dictating fewer fragmentation. On the contrary, the bulky chloride anion is loosely bound to the saccharide, making the chloride ion mobile and thus allowing the creation of multiple charge centers that consist of an ensemble of conformations, which fragment differently, including both glycosidic and cross-ring cleavages. The specific fragmentation pathways leading to the diagnostic ions observed for each sucrose isomer were discussed in our previous studies [31].

**Table 1:** Elution time and corresponding diagnostic ions obtained for the analysis of structural isomers of sucrose (color coded according to MS/MS spectrum)

Sugar	Retention time (min)	Diagnostic ion (*m/z*)
Sucrose α(1 → 2)	15.41	197
Turanose α(1 → 3)	16.84	203
Palatinose α(1 → 6)	17.51	221
Maltulose α(1 → 4)	18.42	263
Leucrose α(1 → 5)	18.26	233, 155, 137
Trehalulose α(1 → 1)	19.60	269

We further analyzed the individual isomers to establish their retention times as well as the fragmentation patterns without the influence of the other isomers. Figure S6 shows the chromatogram of the individual standard sugars with their corresponding MS/MS data summarized in Fig. S7 in negative-ion mode (chloride adduct). It is clearly observed that the results from the individual standard isomers are the same as the data obtained from the mixture of standards in Fig. 3b–g. Similarly, Fig. S8 shows the chromatogram of the mixture of standards with their corresponding MS/MS data in positive-ion mode (sodium adduct).

### Quantitative capabilities

The quantitative ability of the LC-cESI-MS/MS approach for detecting disaccharide isomers was explored in the negative-ion mode by performing calibration studies of the isomers via chloride adduction. In this study, product ion scan was employed. That is to say, the precursor ion of *m/z* 377 was selected for all sugars (*m/z* 389 for internal standard) where all product ions resulting from CID were scanned. The chromatographic peak area of a selected fragment ion of each isomer was plotted against concentration in the range of 0.1 to 1.0 μM. Specific product ions were selected based on the chromatographic signal to noise ratio over the selected concentration range. Thus, for sucrose, the peak area for *m/z* 341 was used while the peak area for *m/z* 179 was used for palatinose, trehalulose, and turanose. Likewise, the peak area for *m/z* 251 was used for leucrose while the peak area for *m/z* 161 was used for maltulose. In the case of the internal standard, the peak area for *m/z* 353 was employed. A least square linear regression was applied for all analytes. In all analyses, the curves were linear with R^2^ ≥ 0.99 except turanose (Fig. S9). To determine the limit of detection (LOD) of each disaccharide positional isomer, a blank solution fortified with the internal standard was analyzed at six replicates and standard deviation (S) calculated. The LOD of each isomer was then determined by multiplying this standard deviation by three (99% confidence level) and dividing by the slope of the calibration curve. In all cases, nanomolar (nM) LODs were obtained as summarized in Table 2**.** The lower LODs obtained demonstrate higher sensitivity for the current method based on chloride adduction, compared with our previous work, which utilized phenylboronic acid chemistry to quantify saccharides [64]. For example, sucrose was detected at LOD 11 nM, which presented one of the sensitive analytes detected on the phenylboronic acid LC–MS/MS platform. The LOD of the same sucrose analyte was 15 × better on our current LC–MS/MS platform utilizing chloride adduction (LOD 0.7 nM). Palatinose registered the lowest LOD of 0.48 nM, which corresponds to 480 fmol absolute amount in the 1 µL injected volume. Intra-day variability for each analyte is summarized in Table S1 in terms of relative standard deviation, which was observed to be < 10% except for the cases of maltulose and palatinose. We further compared the sensitivity of our chloride adduction method in negative-ion mode to the commonly used sodium adduct formation analyzed in positive-ion mode. Here, we performed calibration studies for all the six isomers (Fig. S10). The process was similar to the method applied for chloride adduction, except that a commercial heated electrospray ionization (HESI) source was used for this positive-ion mode analysis. The LODs obtained are provided in Table 2, which registered values 3–4 × higher than that of chloride adduction, indicating lower sensitivity for the sodium adduct detection for the selected isomers. In addition to the high sensitivity, the chloride adduction is found to differentiate closely related isomers more readily than the sodium adducts [31, 65].

**Table 2:** Calibration and limits of detection for disaccharide isomers

Isomer	Chloride adducts			Sodium adducts		
	Identifier for Calibration (*m/z*)	LOD (nM)	R^2^	Identifier for Calibration (*m/z*)	LOD (nM)	R^2^
Sucrose	341	0.70	0.9991	203	2.90	0.9943
Turanose	179	0.93	0.9805	203	1.67	0.9566
Palatinose	179	0.48	0.9979	275	0.28	0.9970
Maltulose	161	0.68	0.9959	347	2.68	0.9998
Leucrose	251	1.10	0.9992	203	0.49	0.9919
Trehalulose	179	0.79	0.9993	203	2.27	0.9990

### Analysis of sucrose isomers in complex honey samples

**Fig. 4:**
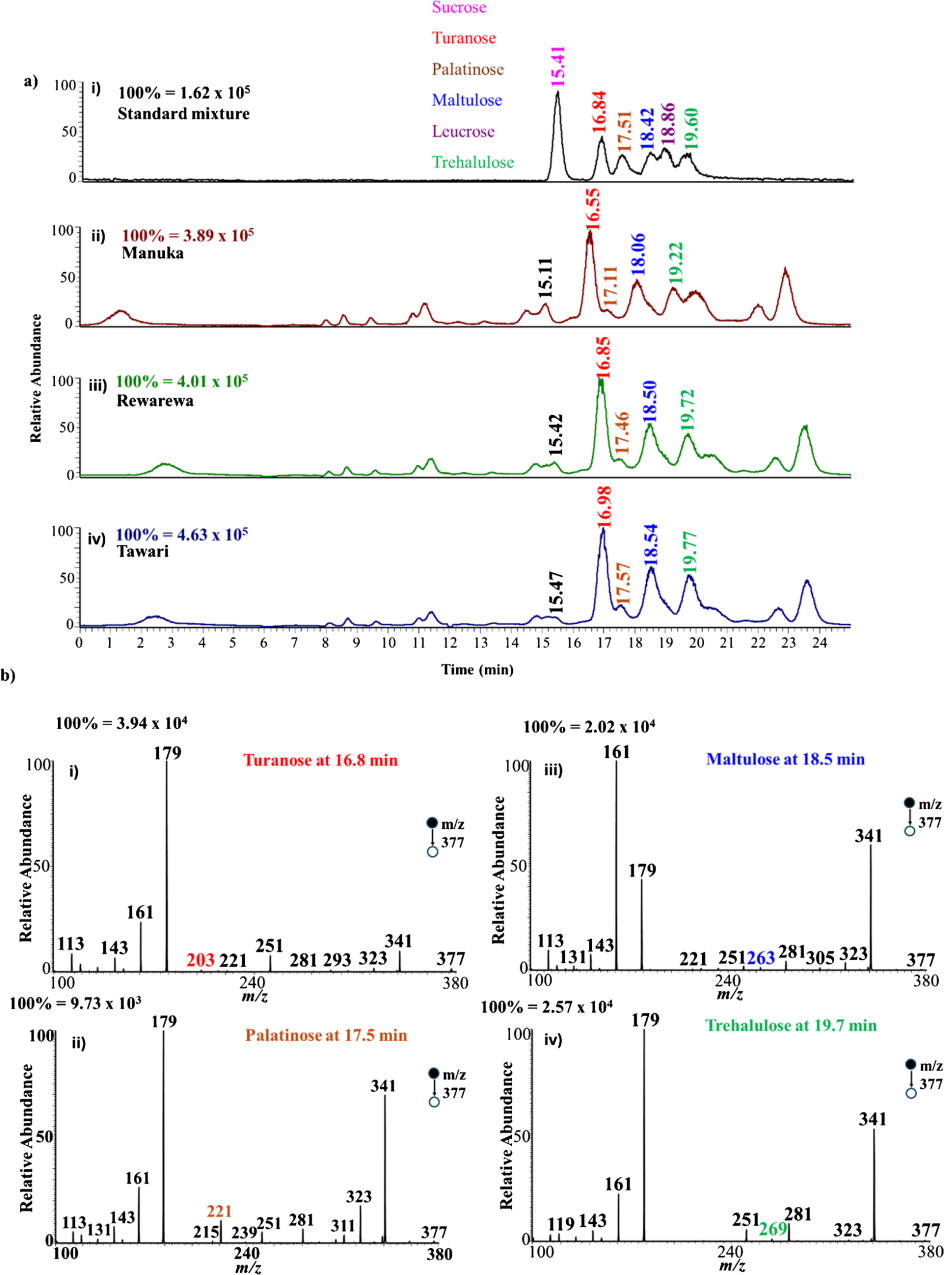
**a** Chromatograms obtained for (i) standard mixture of isomers of sucrose, (ii) Manuka honey, (iii) Rewarewa honey, and (iv) Tawari honey samples. **b** Negative-ion mode MS/MS product ion spectra for four sucrose isomers identified in the Rewarewa honey sample at specified elution time: (i) turanose at 16.8 min, (ii) palatinose at 17.5 min, (iii) maltulose at 18.5 min*,* and (iv) trehalulose at 19.7 min

As stated earlier, honey contains abundant amounts of saccharides, where the saccharides content accounts for more than 95% of the chemical composition. Containing different saccharides including mono-, di-, and oligo-saccharides, honey provides the ideal complex mixture for analysis of isomers of different disaccharides. The positional isomers of sucrose, including palatinose, maltulose, turanose, and trehalulose, are reported to be present in honey [2, 66–70]. We purchased Manuka, Rewarewa, and Tawari mono-floral honey samples from Manawa Honey (New Zealand). These honey samples are derived from three distinct tree sources. The Rewarewa honey is obtained from the nectar of the flowers of the Rewarewa tree (*Knightia excelsa*) as the bees feed on them. Manuka and Tawari honey samples are derived from the nectar of the flowers of the Manuka tree (*Leptospermum scoparium*) and Tawari tree (*Lxerba brexinoides*), respectively [2]. Figure 4a shows the chromatogram for the three honey samples analyzed by product ion scan at *m/z* 377. That is, every signal detected has a mass-to-charge ratio of 377. As can be observed, several peaks are seen in the chromatograms with distinct retention times for the honey samples, besides the expected sucrose isomers.

By comparing the chromatograms (Fig. 4a, ii–iv) from the honey samples with that from the standard mixture (Fig. 4a, i), we can confirm the presence of turanose, palatinose, maltulose, and trehalulose in all three honey samples at retention times ~ 16.8, 17.5, 18.5, and 19.7 min, respectively. The corresponding MS/MS spectra derived from one of the honey samples (i.e., Rewarewa) at *m/z* 377 are provided in Fig. 4b, i–iv. Specific diagnostic ions expected for each isomer were detected, including *m/z* 203 for turanose, *m/z* 221 for palatinose, *m/z* 263 for maltulose, and *m/z* 269 for trehalulose. Among these isomers, palatinose (RT 17.11 min) was found to be the least abundant sucrose isomer in all three mono-floral honey samples. On the other hand, turanose (RT 16.84 min) was the most abundant isomer. Interestingly, a similar distribution for palatinose and turanose was recently reported for three poly-floral honey samples, where abundant turanose was detected with palatinose showing low abundance [48, 70]. Based on MS/MS and separation data for the standard mixture analysis (Fig. 4a, i), leucrose (RT 18.86 min) is not present in any of the mono-floral honey samples. Previous studies have also indicated that honey’s saccharase (invertase) enzyme is unable to generate leucrose during the maturation process and hence its absence in naturally occurring honey [15, 71].

**Fig. 5:**
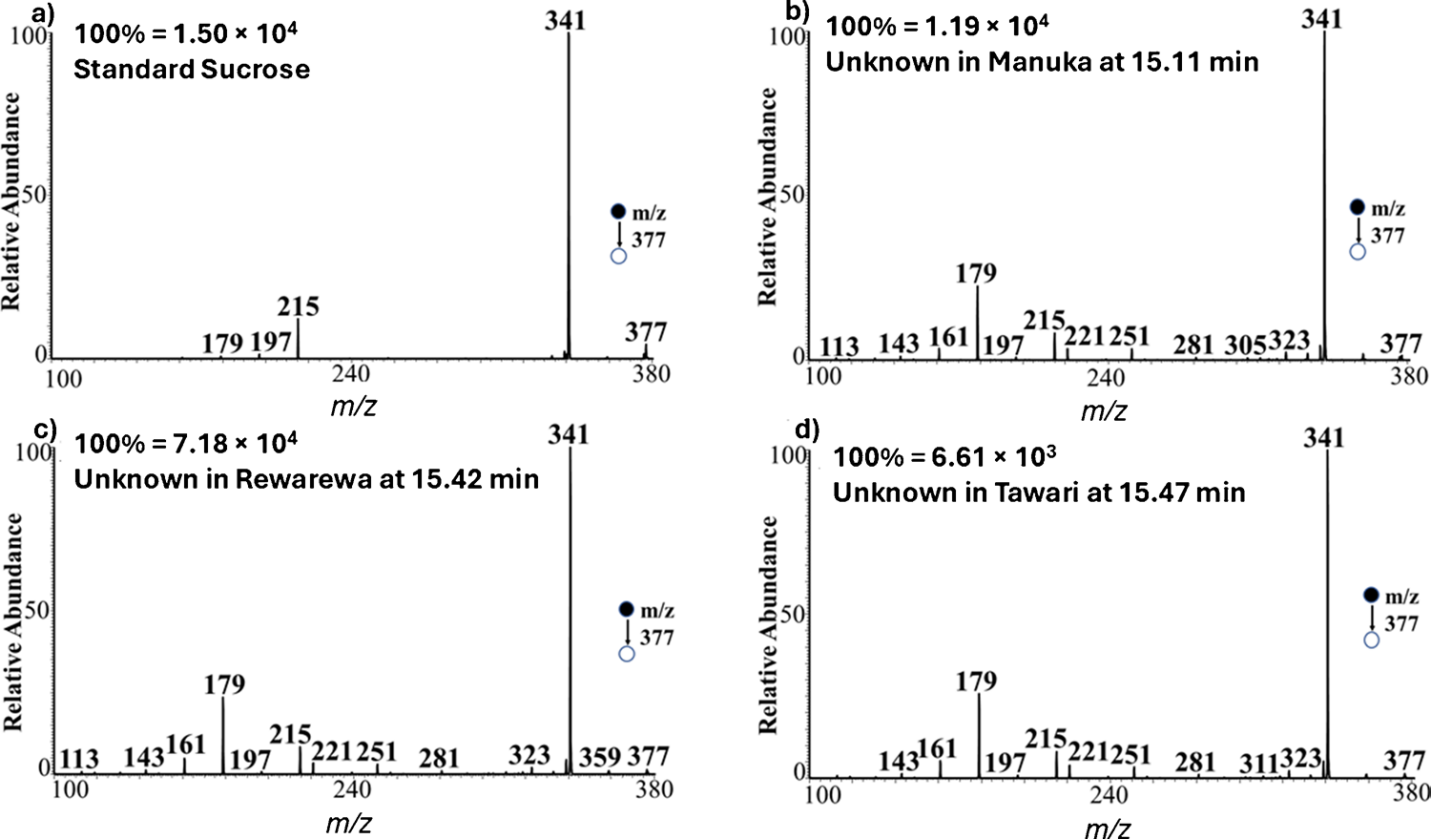
Negative-ion mode tandem MS spectra of **a** standard sucrose and **b**–**d** unknown species in honey samples, as obtained from Fig. 4a at retention times **b** 15.11 min (Manuka), **c** 15.42 min (Rewarewa), and **d** 15.47 min (Tawari) all at *m/z* 377

Based on retention time information only, one may wrongfully assign the peaks at 15.11, 15.42, and 15.47 min observed from Manuka, Rewarewa, and Tawari samples, respectively, (Fig. 4a, ii–iv) to be sucrose. If the slight shifts in retention times are considered as random error (RT differ by 1%), then it makes sense to assign these peaks as to be identical, which can correspond to sucrose (RT 15.41 min). However, the MS/MS data recorded at these retention times indicates a different compound, other than sucrose, is present. Figure 5a shows the MS/MS for standard sucrose, which is compared with the MS/MS data recorded from the three mono-floral honey samples at retention times 15.11, 15.42, and 15.47 min (Fig. 5b–d). It can be observed that the fragmentation pattern of sucrose is different from the pattern observed for the unknown species found in the honey samples. From all experiments conducted on standard sucrose, we observed that the disaccharide fragments to give only a set of few ions during tandem MS. However, tandem MS analyses of the unknown species in the honey samples (Fig. 5b–d) showed a multitude of product ions that differ from those observed for standard sucrose (Fig. 5a). This result represents an example of an instance where two-dimensional data collection is important for the complete identification of saccharide isomers in complex mixture analysis. Therefore, caution must be exercised when quoting the amount of sucrose in honey samples [1]. Based on our data, four isomers of sucrose (i.e., turanose, palatinose, maltulose, and trehalulose) are more abundant in the tested honey samples than sucrose itself, which is not detected in the current study. Although the current work focused on six sucrose isomers, further studies are required to characterize the unknown species, not only the species around retention time 15.41 ± 0.06 min but also species detected at 7.96, 8.53, 9.42, 10.77, 11.17, 14.48, 19.96, 22.0, and 22.88 min. Overall, nine (9) major species were detected in all the three mono-floral honey samples, in addition to some five minor species with retention time below 12 min. The orthogonal LC–MS presented here, with informative collision-induced dissociation data, enabled four of the nine major species to be confirmed, excluding known disaccharides such as sucrose and leucrose. Our approach exploits inherent structural differences and requires no additional hardware or multiple collision energy, making it simpler and more compatible with routine LC–MS/MS workflows compared with ER-MS. While IMS remains a powerful complementary technique, our approach offers practical advantages in accessibility and ease of implementation.

### Classification of honey samples using LC-cESI-MS/MS

Following the successful analysis of sucrose isomers via online microdroplet-based Cl^−^ adduction on the LC–MS/MS platform, we further explored the characterization of these isomers using principal component analysis (**PCA**), employing the MetaboAnalyst online software. Previously, honey samples have been classified using amino acids, DNA metabarcoding, phenolics, flavonoids, and carbohydrates [2, 4, 7, 8]. In this study, LC–MS/MS chromatograms were extracted from the region corresponding to the elution of the four identified sucrose isomers—turanose, palatinose, maltulose, and trehalulose—in honey samples. These chromatograms were saved as.csv files in Microsoft Excel for subsequent analysis. The data were then uploaded to MetaboAnalyst, where they were normalized using the sum method followed by auto-scaling. LC–MS/MS data, collected in both negative- (chloride adduct) and positive- (sodium adduct) ion modes, were used for comparison.

**Fig. 6:**
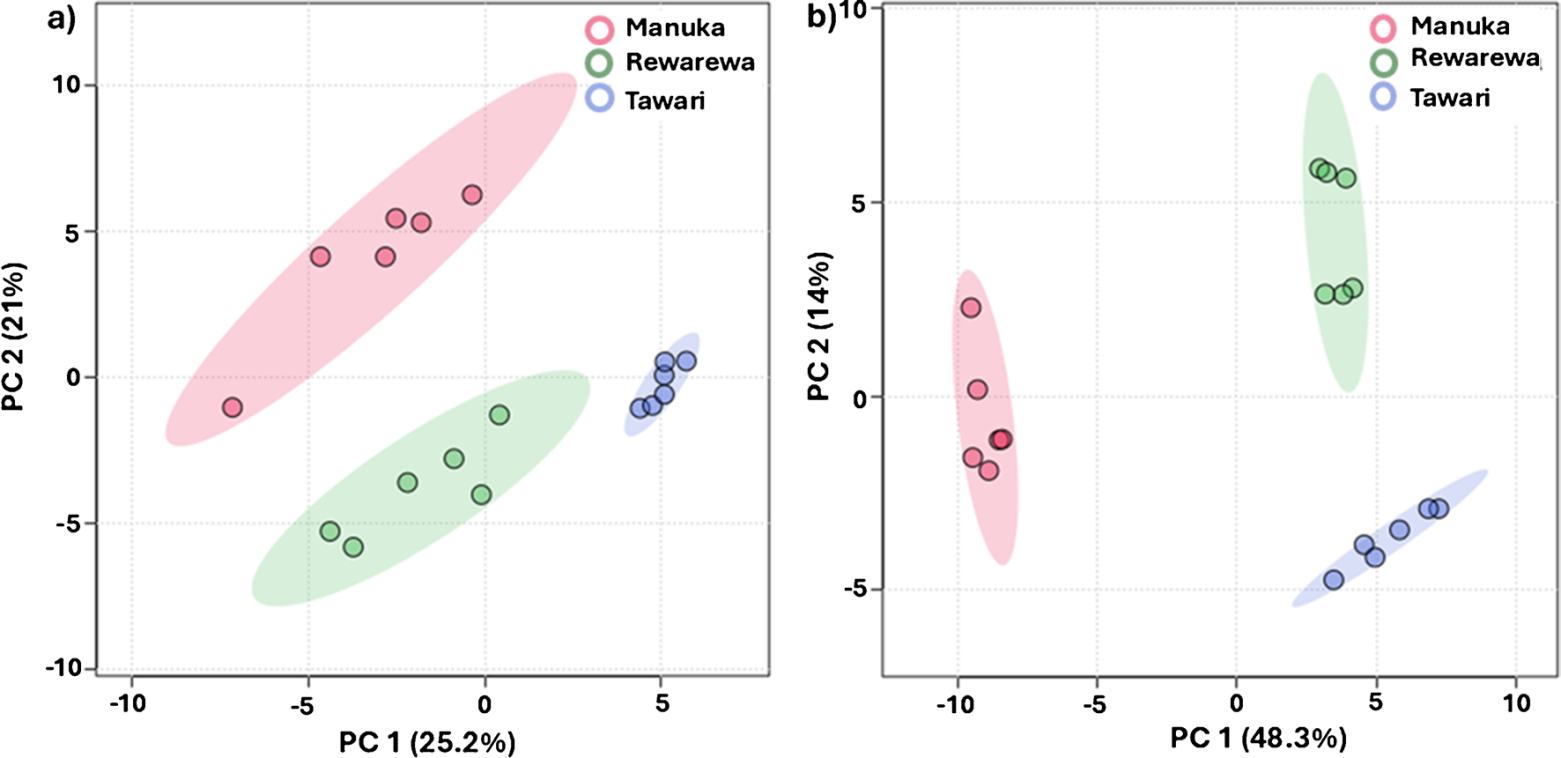
PCA scores plots obtained for **a** negative-ion mode tandem MS and **b** positive-ion mode tandem MS, of sucrose isomers. Two first-component scores (PC1 and PC2) are plotted for six technical replicates of Manuka, Rewarewa, and Tawari honey samples

The PCA, shown in Fig. 6, presents the score plot of PC1 versus PC2 derived from the different honey samples. Interestingly, both ionization modes effectively classified all the honey samples analyzed, with the positive-ion mode (62% variance) slightly outperforming the negative-ion mode (46% variance). Further analysis was conducted using a heatmap (t-test/ANOVA), which revealed a strong correlation between specific ions generated during tandem MS and the honey sample identity. Notably, diagnostic ions, such as *m/z* 221 for palatinose and *m/z* 263 for maltulose, were instrumental in successfully grouping the honey samples (Fig. S11). In contrast, common ions (e.g., *m/z* 203, 365, and 275) present in sucrose isomers were primarily responsible for classification in the positive-ion mode (Fig. S12). These findings suggest that both the intensity and identity of the ions are crucial for classifying honey species using the negative-ion mode, while in positive-ion mode, ion intensity alone determines the separation of honey samples. Collectively, despite the honey samples exhibiting similar sucrose isomer profiles, the application of PCA and heatmap analysis proved to be effective in distinguishing and classifying different types of mono-floral honey.

## Conclusions

In conclusion, this study underscores the efficacy of the LC-cESI-MS/MS platform in analyzing and differentiating positional isomers of sucrose in complex mixtures with high sensitivity and specificity. By employing chloride adduction, we achieved a robust two-dimensional analysis that integrates chromatographic retention time with MS/MS diagnostic ions obtained using the widely available ion activation method, collision-induced dissociation, and thereby eliminating ambiguities in isomer characterization. We identified palatinose α(1 → 6) to be the least abundant sucrose isomer in all three mono-floral honey samples, while turanose α(1 → 3) was observed to be the most abundant isomer. The presence of sucrose α(1 → 2) could not be confirmed in any of the mono-floral honey samples. Thus, the LC-cESI-MS/MS methodology demonstrated its practicality and versatility by successfully identifying sucrose isomers in diverse honey samples, offering insights into their specific disaccharide composition. These findings emphasize the potential of our platform for broader applications in complex carbohydrate analysis and its capacity to facilitate rapid, sensitive, and detailed molecular characterization of saccharide isomers, paving the way for advancements in food chemistry, analytical sciences, and beyond. Further studies are required to characterize the other disaccharide species detected in the honey samples, which can be accomplished by including more standards such as both alpha and beta configurational isomers. The specific amount of the identified isomers in the honey samples will be quantified in subsequent studies.

## Supplementary Information

Below is the link to the electronic supplementary material.

Supplementary Material 1 (PDF 1.54 MB)

## Data Availability

The authors confirm that the data supporting the findings of this study are available within the article and/or its supplementary materials.
