# Supplementary material for: Liquid chromatography-mass spectrometry approach for characterizing sucrose isomers in complex mono-floral honey
Source: Anal Bioanal Chem. 2025 Jul 9;417(20):4709–22. doi: 10.1007/s00216-025-05988-9 (PMC12325442; doi:10.1007/s00216-025-05988-9)
Supplement: Supplementary file 1 — Supplementary Material 1 (PDF 1.54 MB) [file 216_2025_5988_MOESM1_ESM.pdf]

SUPPORTING INFORMATION

Liquid Chromatography-Mass Spectrometry Approach for Characterizing Sucrose Isomers in Complex Mono-Floral Honey

*Enoch Amoah, Santosh Raman Acharya, Ayesha Seth, and Abraham K. Badu-Tawiah**

Department of Chemistry and Biochemistry, The Ohio State University, Columbus, OH 43210, USA

*Correspondence to Dr. Abraham K. Badu-Tawiah

100 W. 18th Avenue, Columbus OH, 43210

Email: badu-tawiah.1@osu.edu

Tel.: 614-292-4276

Fax: (614) 292-1685

Table of Contents

1. ***Study of Voltage Effect on Sugar-Chloride Adduct Formation using cESI Platform........ 3***

2. ***Optimization of Reagent Flow Rate for Chloride-Sucrose Adduction.............................. 4***

3. ***Effect of Chloride concentration in Adduction of Sucrose... 4***

4. ***Effect of Column Flow Rate on Retention Time... 5***

5. ***Effect of Column Temperature on Retention Time... 6***

6. ***LC-MS/MS Analysis of Standard Individual Isomers... 7***

7. ***Tandem MS Analysis of Standard Individual Isomers.. 8***

8. ***LC-MS/MS Analysis of Isomers in Mixture in Positive ion mode................................... 9***

9. ***Calibration Plots of Chloride Adducted Disaccharides 10***

10. ***Calibration Plots of Sodium Adducted Disaccharides... 11***

11. ***Intra-day repeatability of sucrose isomers 12***

12. ***Heat Maps for negative and positive- ion mode of analysis... 12***

1. Study of Voltage Effect on Sugar-Chloride Adduct Formation using cESI Platform

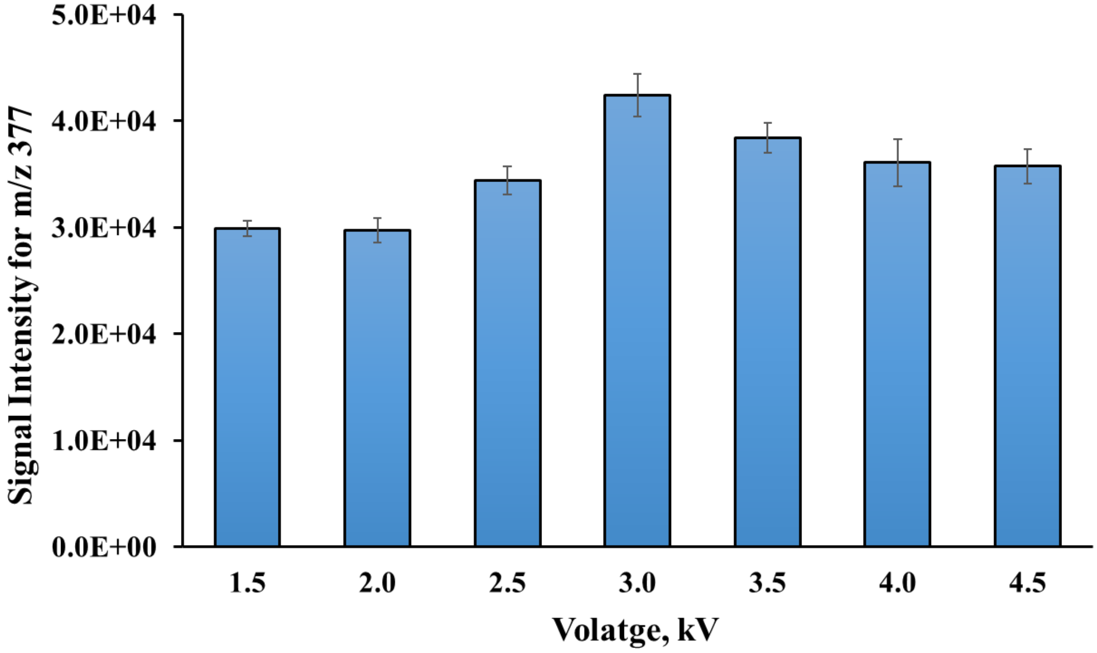

Figure S1. Effect of voltage on chloride adduction of sucrose (100 μM). Error bars represent standard deviation of five replicates.

2. Optimization of Reagent Flow Rate for Chloride-Sucrose Adduction

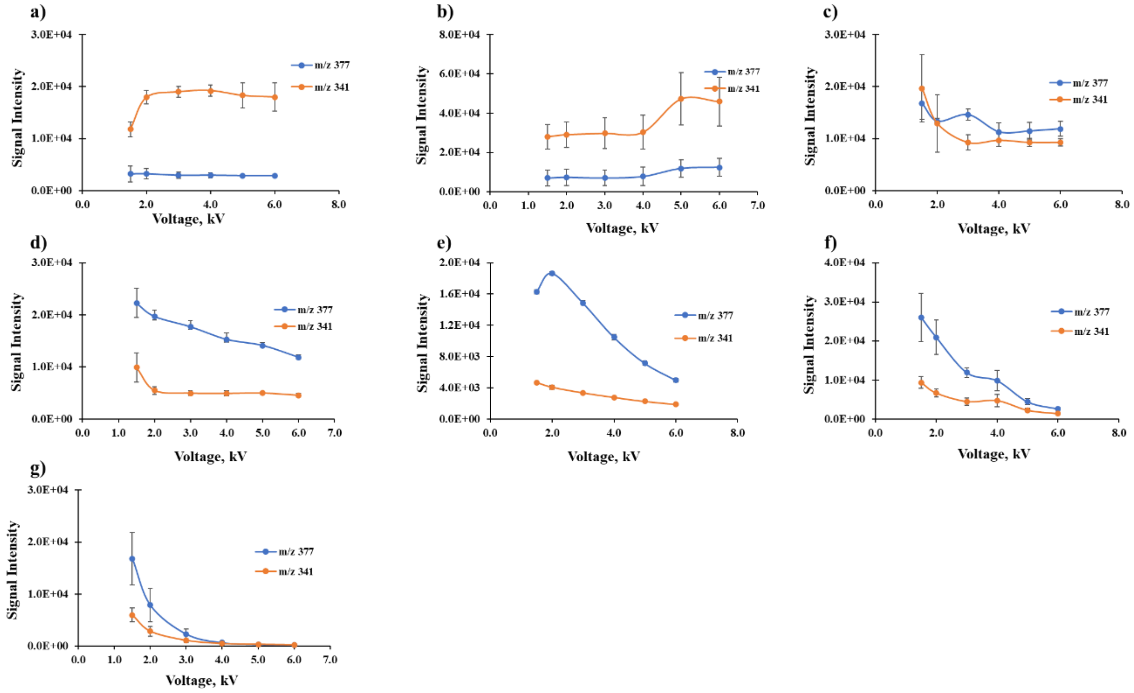

Figure S2. Effect of reagent flow rate on chloride adduction relative to deprotonated sucrose at **a)** 5 **b)** 10 **c)** 20 **d)** 30 **e)** 40 **f)** 50 and **g)** 60 µL/min. Error bars represent standard deviation of three replicates.

3. Effect of Chloride concentration in Adduction of Sucrose

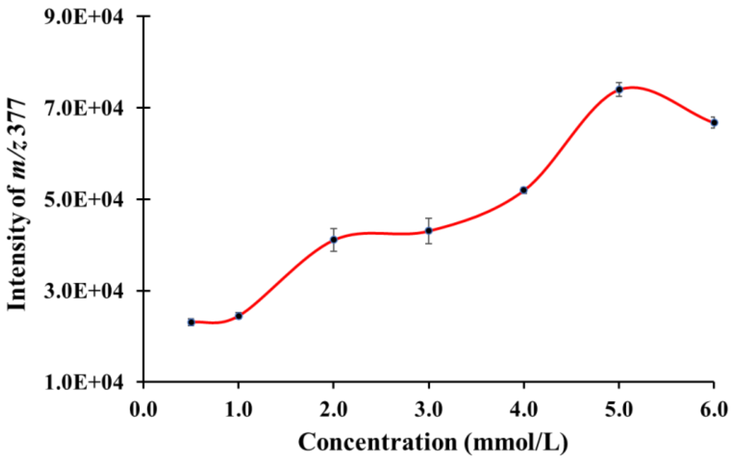

Figure S3. Plot showing effect of chloride concentration on sucrose adduction. Error bars represent standard deviation of five replicates.

4. Effect of Column Flow Rate on Retention Time

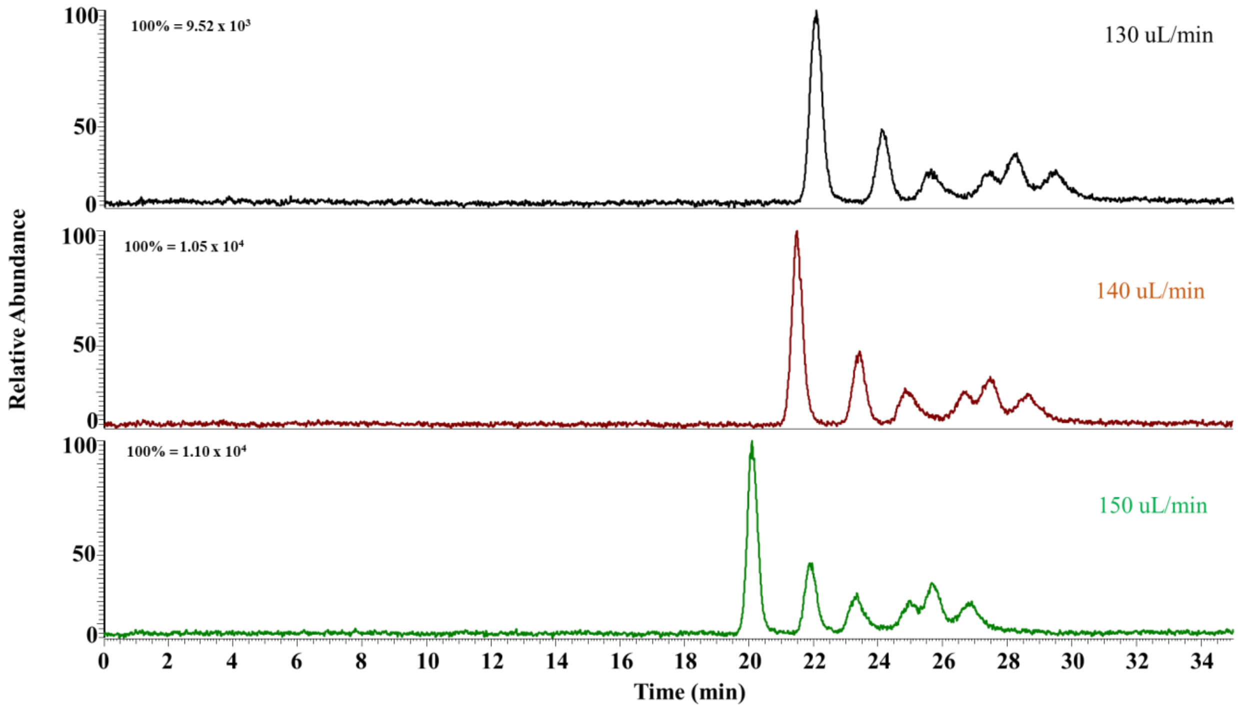

Figure S4. Chromatograms showing separation of mixture of sucrose isomers (2μM) at various flow rates (μL/min).

5. Effect of Column Temperature on Retention Time

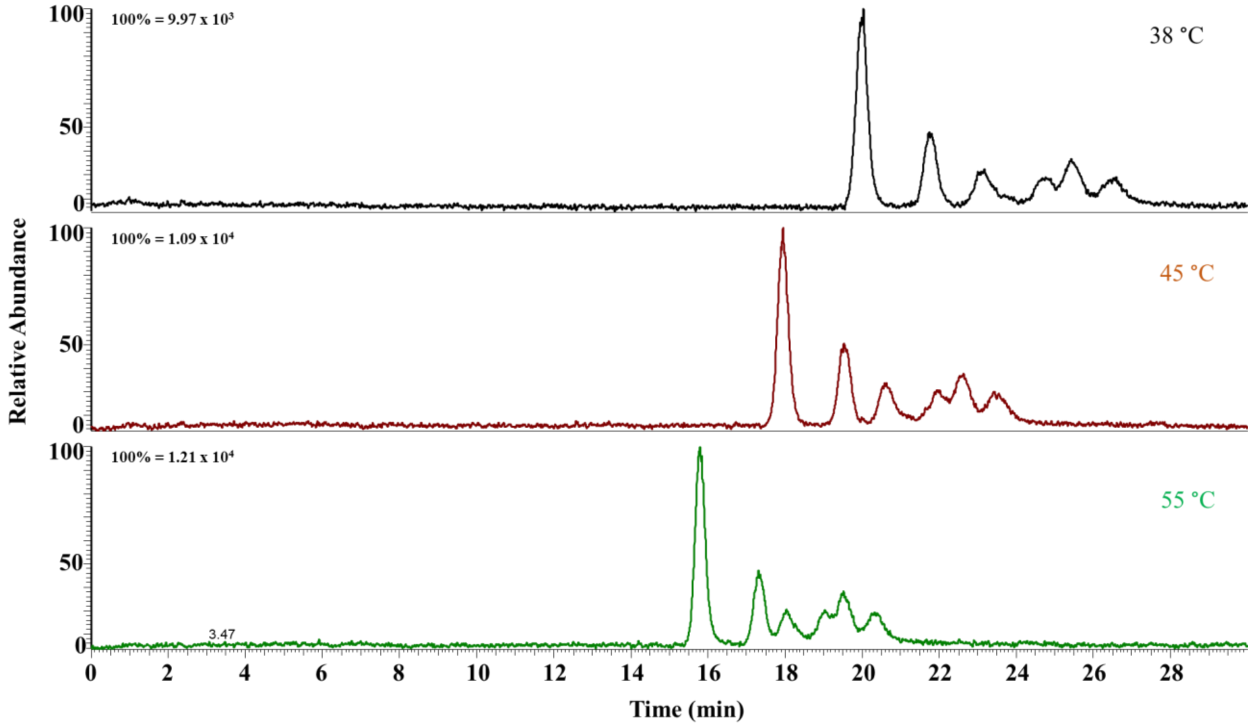

Figure S5. Chromatograms showing separation of mixture of sucrose isomers (2µM) at various column temperatures (˚C).

6. LC-MS/MS Analysis of Standard Individual Isomers

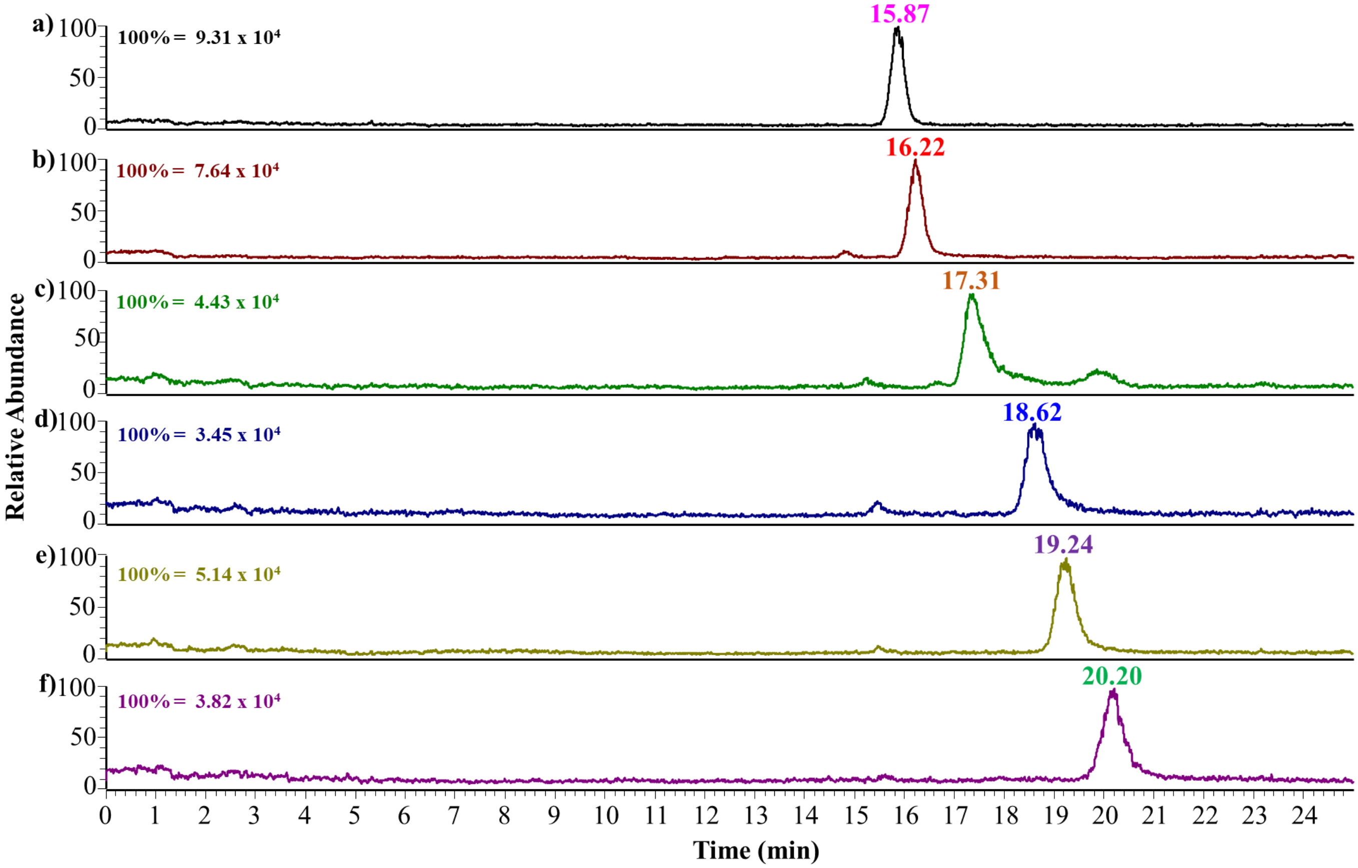

Figure S6. Individual chromatogram for **a)** Sucrose **b)** Turanose **c)** Palatinose **d)** Maltulose **e)** Leucrose **f)** Trehalulose each at concentration of 2μM.

7. Tandem MS Analysis of Standard Individual Isomers

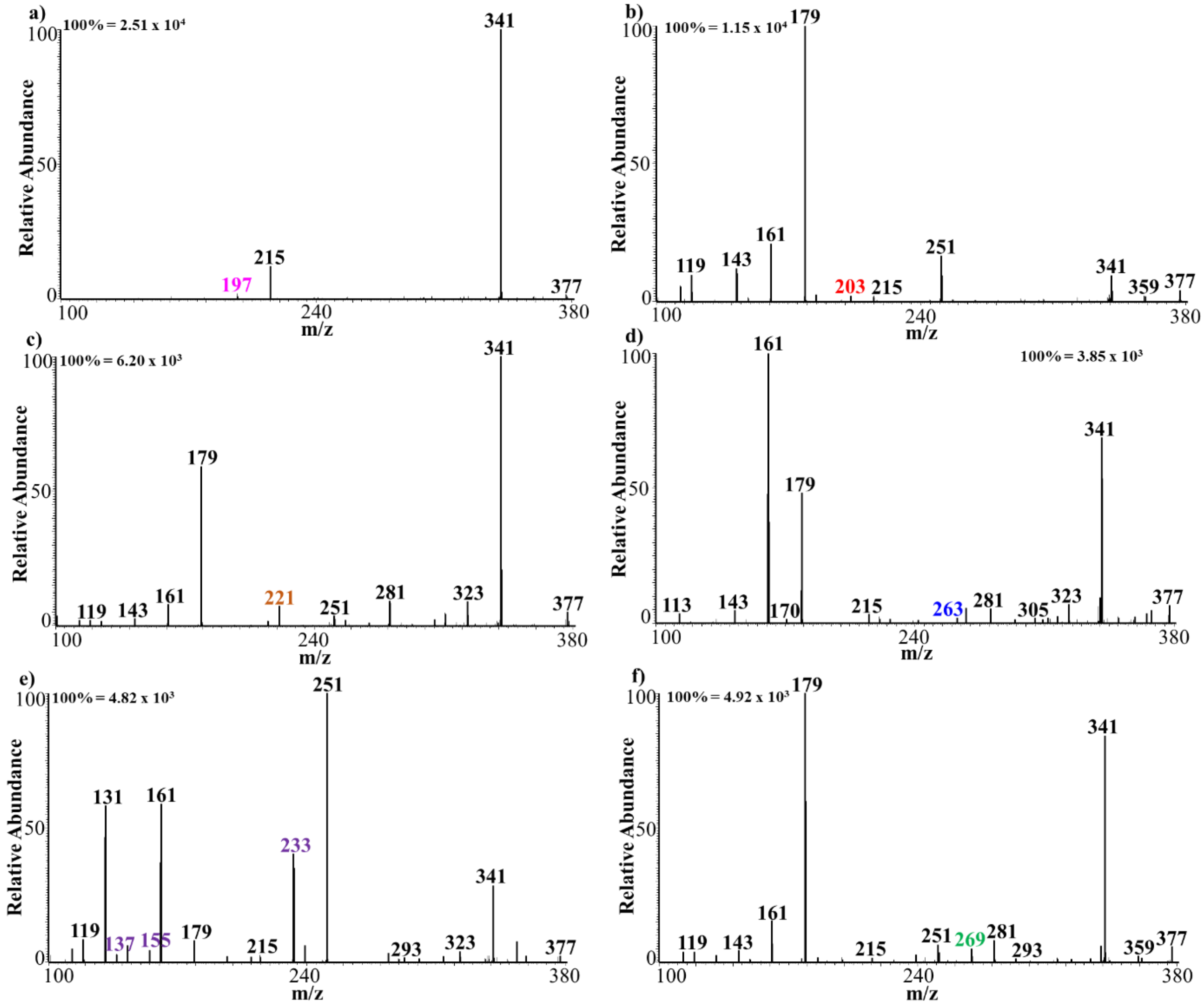

Figure S7. Negative-ion mode product ion spectra for each of the isomers of sucrose (2µM) in the standard chromatogram, **a)** Sucrose **b)** Turanose **c)** Palatinose **d)** Maltulose **e)** Leucrose **f)** Trehalulose, all at *m/z* 377. Different color indicates the diagnostic ions for specific sucrose isomer upon MS/MS.

8. LC-MS/MS Analysis of Isomers in Mixture in Positive ion mode

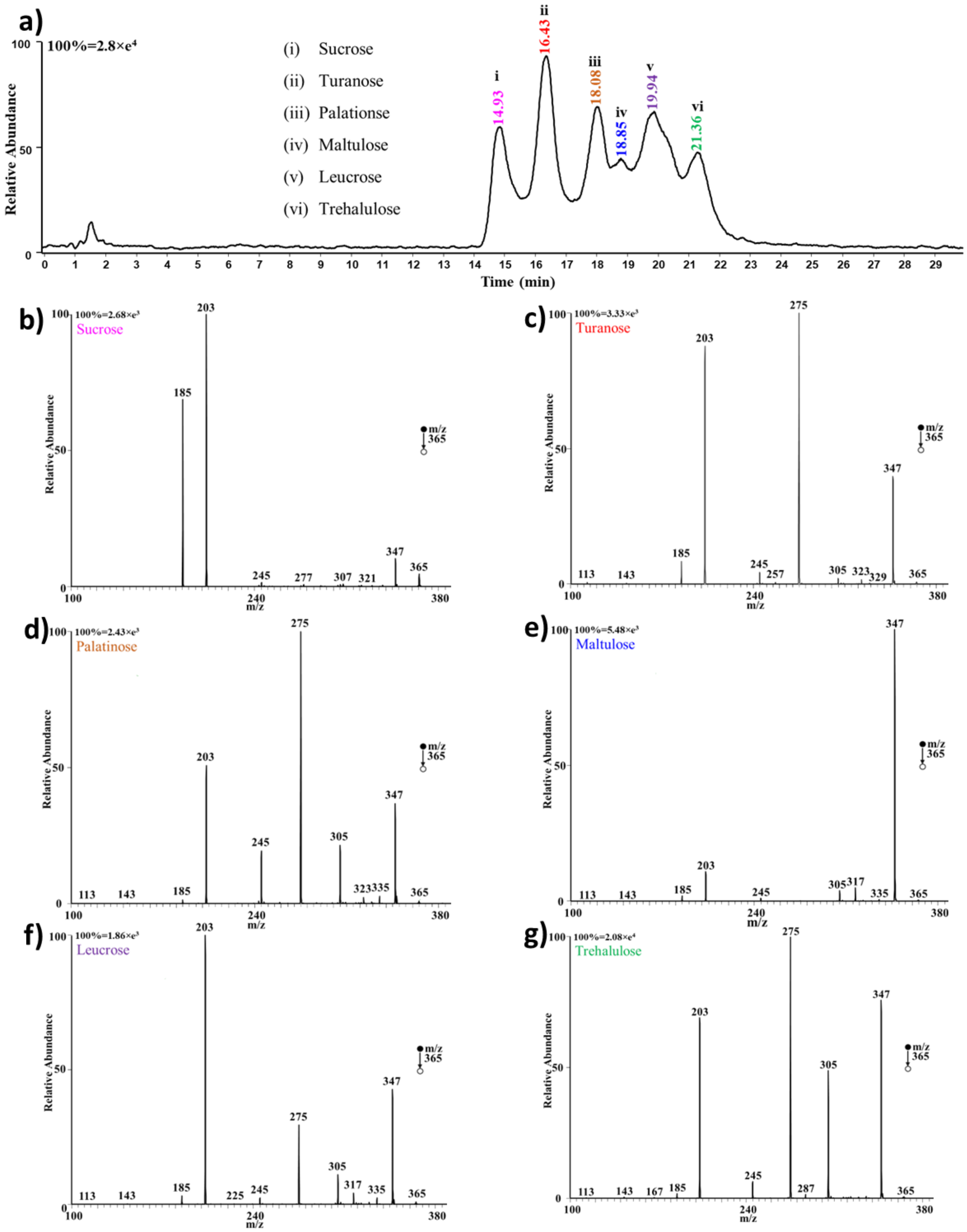

Figure S8. a) Chromatogram generated using LC-MS/MS product ion scanning with sodium adduction **b-g)** Positive-ion mode product ion spectra for isomers of sucrose (2µM) in the standard chromatogram, all at *m/z* 365.

9. Calibration Plots of Chloride Adducted Disaccharides

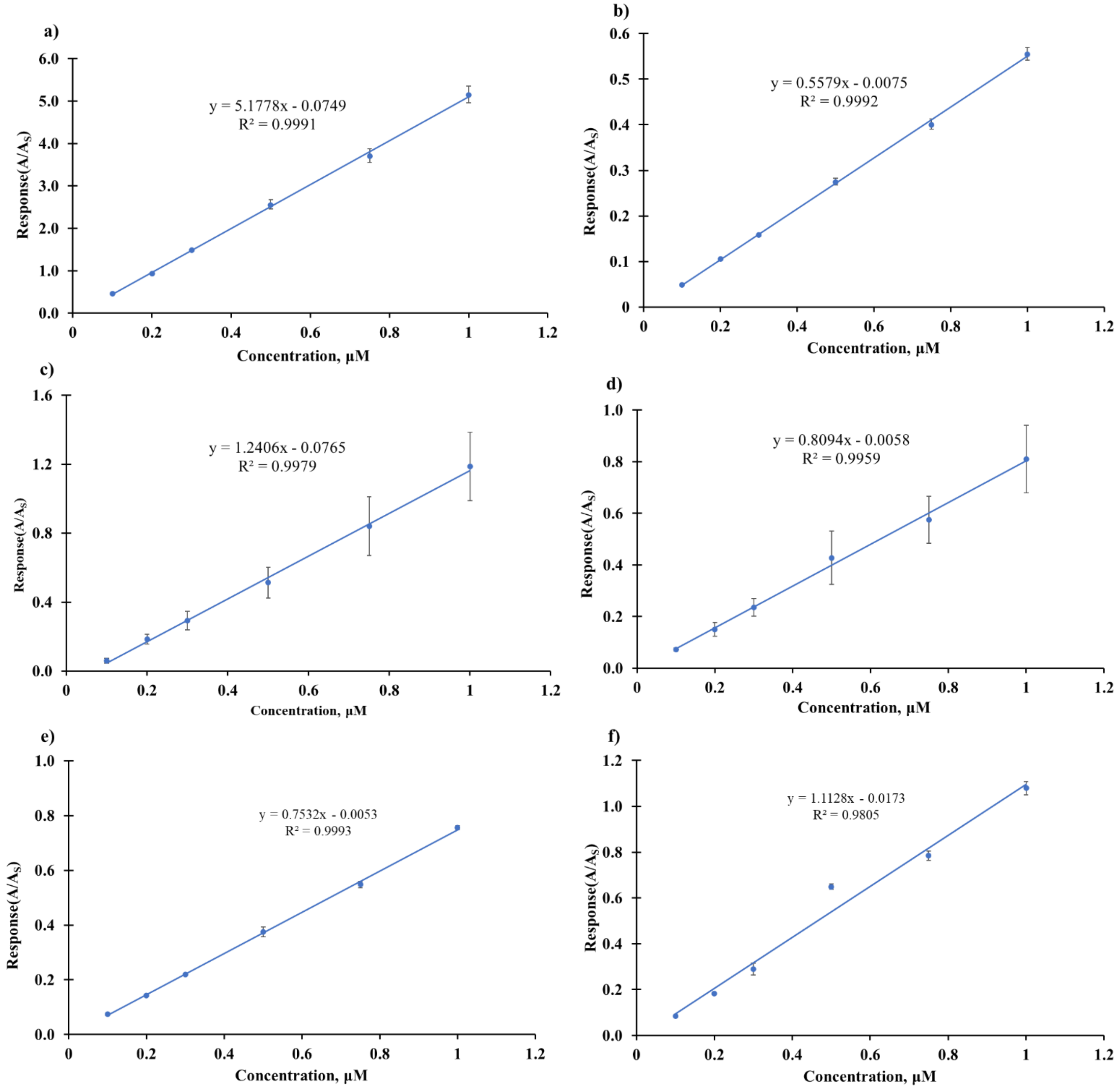

Figure S9. Negative-ion mode chloride adducted calibration plot for **a)** Sucrose **b)** Leucrose **c)** Palatinose **d)** Maltulose **e)** Trehalulose and **f)** Turanose.

10. Calibration Plots of Sodium Adducted Disaccharides

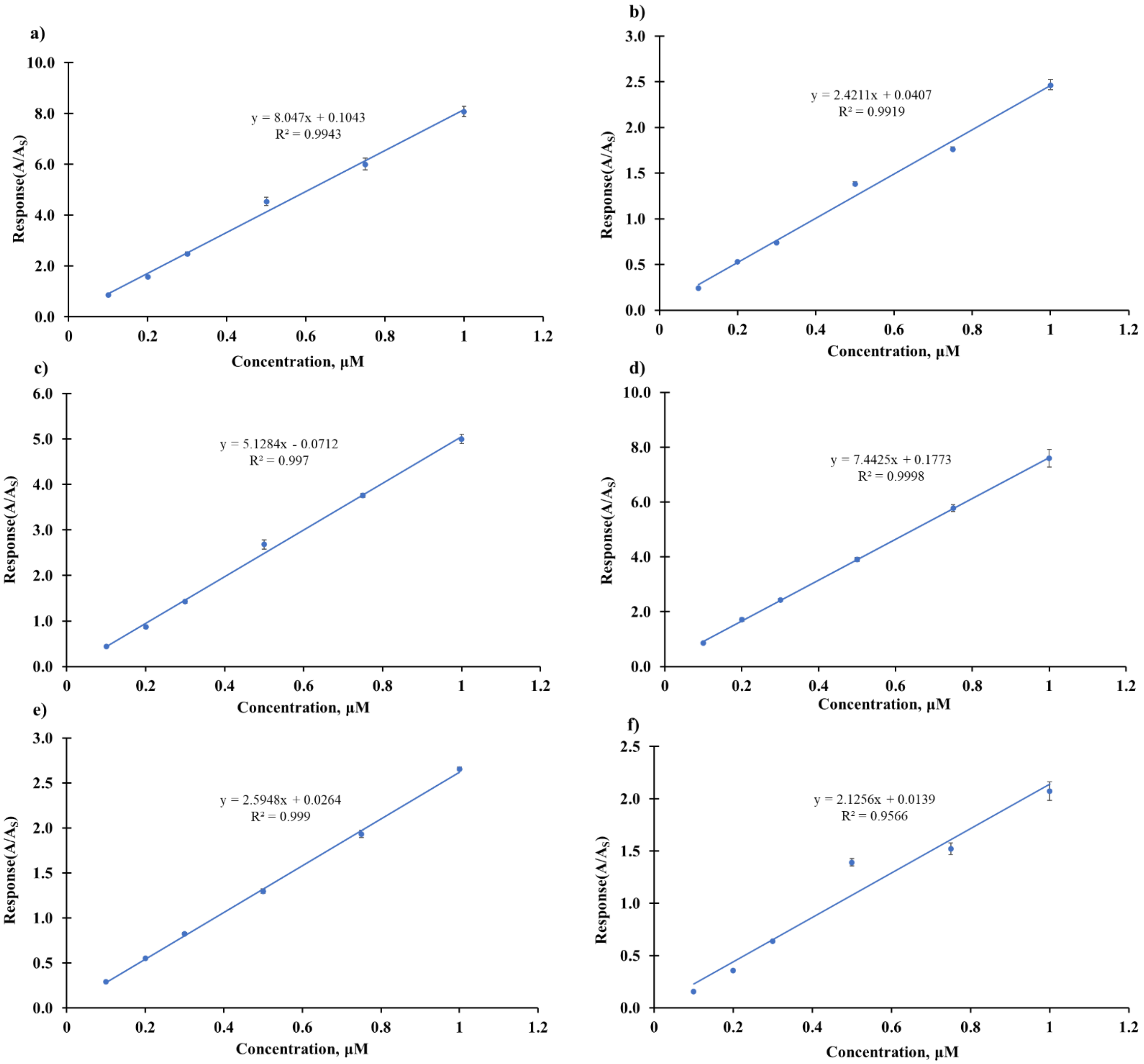

Figure S10. Positive-ion mode sodium adducted calibration plot for **a)** Sucrose **b)** Leucrose **c)** Palatinose **d)** Maltulose **e)** Trehalulose and **f)** Turanose.

11. Intra-day repeatability of sucrose isomers

Table S1. Relative standard deviation of sucrose isomers (n=4) at two different concentrations.

Concentration (μM)	RSD (%)					
	Sucrose	Turanose	Palatinose	Maltulose	Leucrose	Trehalulose
0.1	2.5	2.0	21.9	14.6	3.4	6.8
0.5	8.7	3.7	20.7	9.1	5.1	9.5

12. Heat Maps for negative and positive- ion mode of analysis

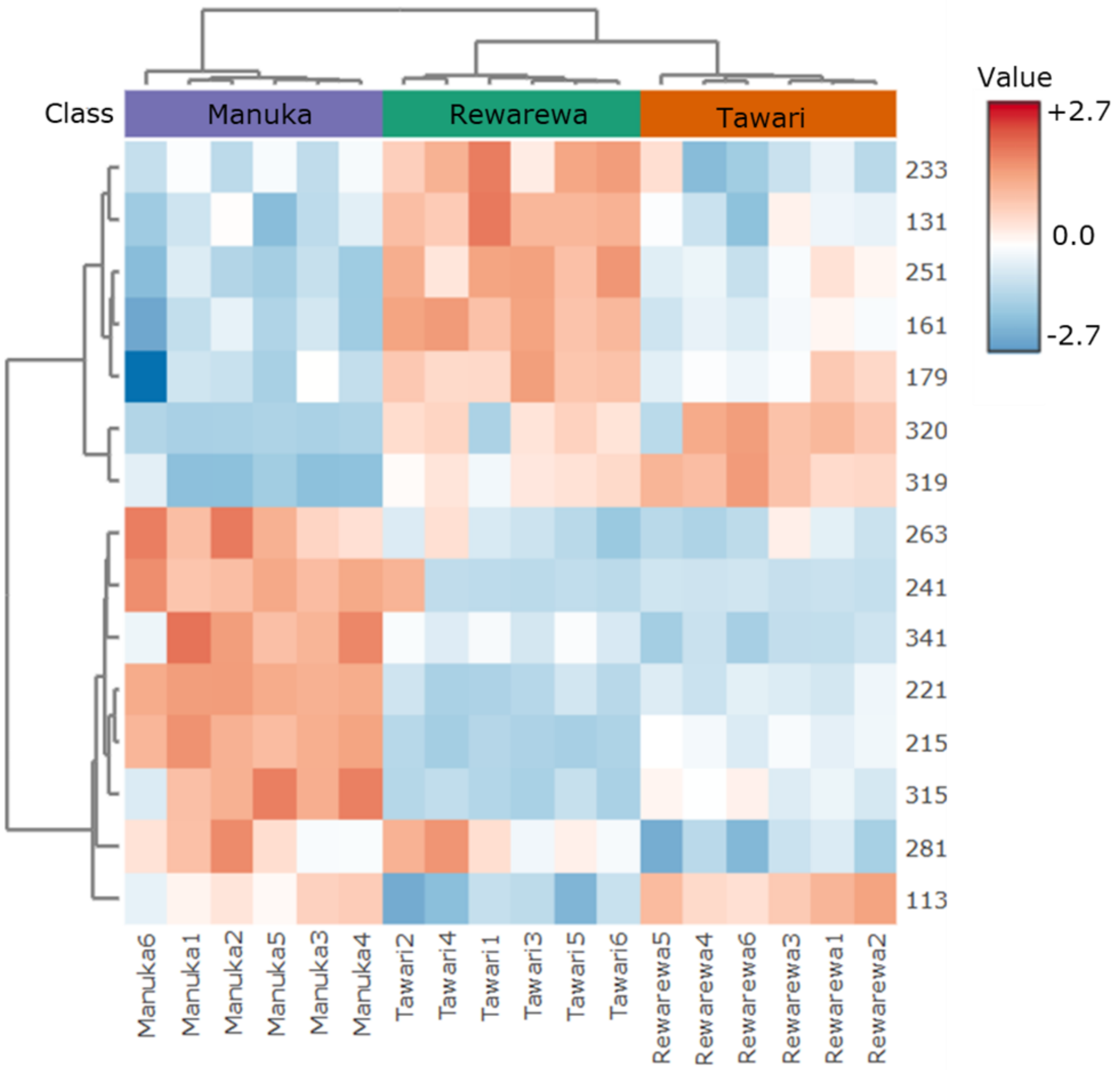

Figure S11. Heatmap results obtained for negative-ion mode tandem MS for sucrose isomers identified in all the honey samples (i.e; Turanose, Palatinose, Maltulose and Trehalulose).

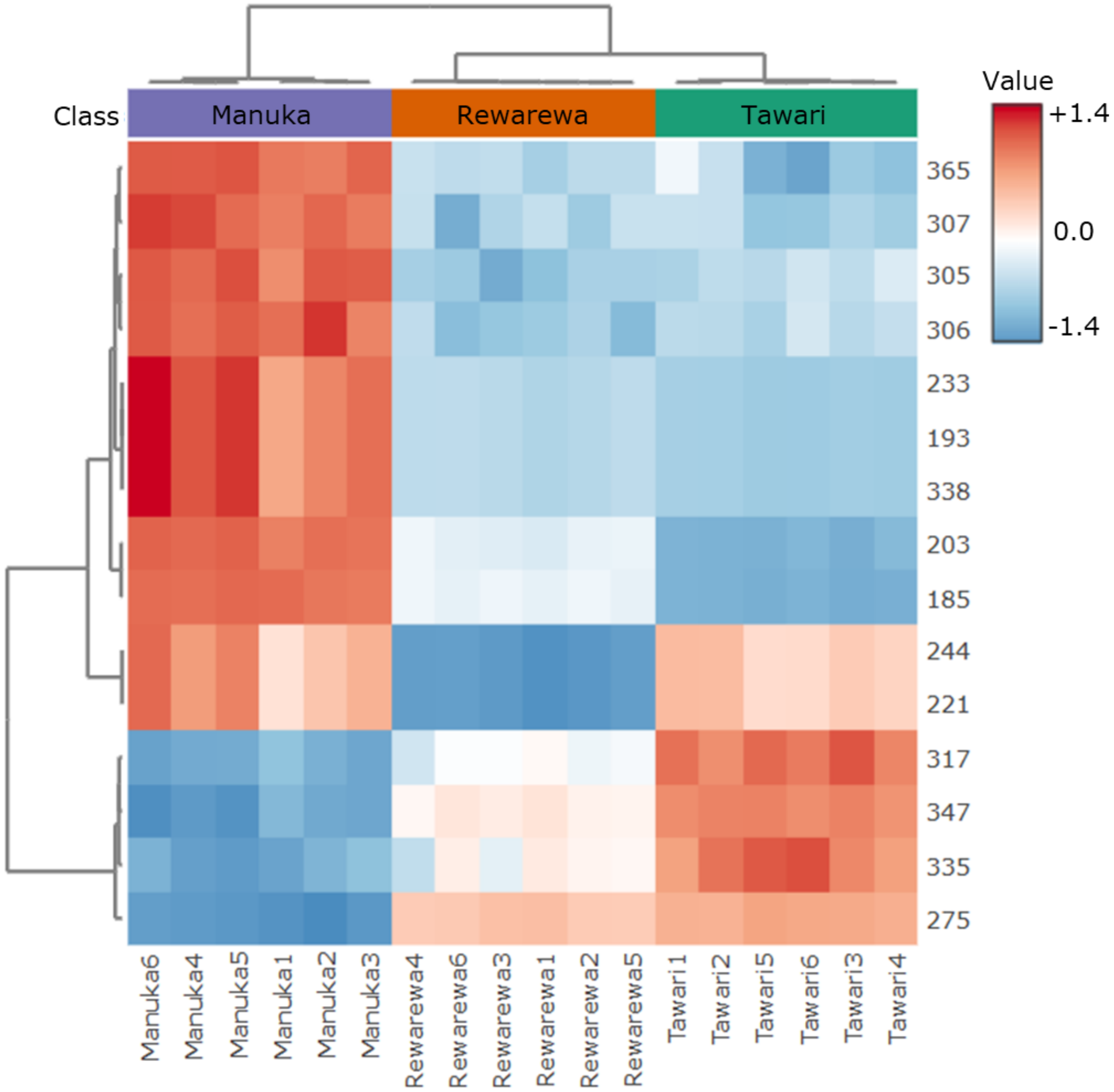

Figure S12. Heatmap results obtained for positive-ion mode tandem MS for sucrose isomers identified in all the honey samples (i.e; Turanose, Palatinose, Maltulose and Trehalulose).
